# Inter-population differences in salinity tolerance of adult wild Sacramento splittail: osmoregulatory and metabolic responses to salinity

**DOI:** 10.1093/conphys/coaa098

**Published:** 2020-12-10

**Authors:** Christine E Verhille, Theresa F Dabruzzi, Dennis E Cocherell, Brian Mahardja, Fred Feyrer, Theodore C Foin, Melinda R Baerwald, Nann A Fangue

**Affiliations:** 1 Department of Wildlife, Fish, and Conservation Biology, University of California, 1 Shields Ave., Davis, CA 95616, USA; 2 Department of Ecology, Montana State University, 310 Lewis Hall ,Bozeman, MT 59717, USA; 3 Biology Department, Saint Anselm College, 100 Saint Anselm Drive, Manchester, NH 03102, USA; 4 United States Fish and Wildlife Service, Department of the Interior, Delta Juvenile Fish Monitoring Program, 850 South Guild Ave, Suite 105, Lodi, CA, USA; 5 California Water Science Center, U.S. Geological Survey, 6000 J St., Sacramento, CA 95819-6129, USA; 6 Department of Plant Sciences, University of California, 1 Shields Ave., Davis, CA 95616, USA; 7 Division of Environmental Services, California Department of Water Resources, 3500 Industrial Boulevard, West Sacramento, CA 95691, USA

**Keywords:** California, cyprinid, metabolism, osmoregulation, salinity, splittail

## Abstract

The Sacramento splittail (*Pogonichthys macrolepidotus*) is composed of two genetically distinct populations endemic to the San Francisco Estuary (SFE). The allopatric upstream spawning habitat of the Central Valley (CV) population connects with the sympatric rearing grounds via relatively low salinity waters, whereas the San Pablo (SP) population must pass through the relatively high-salinity Upper SFE to reach its allopatric downstream spawning habitat. We hypothesize that if migration through SFE salinities to SP spawning grounds is more challenging for adult CV than SP splittail, then salinity tolerance, osmoregulatory capacity, and metabolic responses to salinity will differ between populations. Osmoregulatory disturbances, assessed by measuring plasma osmolality and ions, muscle moisture and Na^+^-K^+^-ATPase activity after 168 to 336 h at 11‰ salinity, showed evidence for a more robust osmoregulatory capacity in adult SP relative to CV splittail. While both resting and maximum metabolic rates were elevated in SP splittail in response to increased salinity, CV splittail metabolic rates were unaffected by salinity. Further, the calculated difference between resting and maximum metabolic values, aerobic scope, did not differ significantly between populations. Therefore, improved osmoregulation came at a metabolic cost for SP splittail but was not associated with negative impacts on scope for aerobic metabolism. These results suggest that SP splittail may be physiologically adjusted to allow for migration through higher-salinity waters. The trends in interpopulation variation in osmoregulatory and metabolic responses to salinity exposures support our hypothesis of greater salinity-related challenges to adult CV than SP splittail migration and are consistent with our previous findings for juvenile splittail populations, further supporting our recommendation of population-specific management.

## Teaser

Genetically distinct populations of the Sacramento splittail, a minnow endemic to the San Francisco Estuary, exhibit different tolerances and osmoregulatory and metabolic responses to salinity as adults. Inter-population variation corresponds with that of juveniles and known salinity habitat differences, suggesting some degree of local adaptation.

## Introduction

Sacramento splittail (*Pogonichthys macrolepidotus*) is a minnow endemic to California’s San Francisco Estuary (SFE) and its associated rivers and tributaries. This sole extant member of the *Pogonichthys* genus (Moyle, 2002; Moyle *et al*., 2004) has recently been resolved into two genetically distinct populations, referred to as the San Pablo (SP) and Central Valley (CV) populations (Baerwald *et al*., 2007, 2008; Mahardja *et al*., 2015) based on allopatric spawning habitat locations (Feyrer *et al*., 2015). Otolith strontium signatures of wild-captured fish have shown age-0 SP splittail to experience greater water salinities than the equivalent CV life stages (Feyrer *et al*., 2005, 2007, 2010). In our previous studies, modest interpopulation variation in juvenile splittail physiological responses (Verhille *et al*., 2016) to salinity was reflected in divergent transcriptomic responses indicative of improved plasticity, especially for gill remodelling, in the SP population (Jeffries *et al*., 2019; Mundy *et al*., 2020). This variation corresponded with previously reported strontium signature findings, suggesting local adaptation of juvenile SP splittail to its higher-salinity spawning habitat. However, high-salinity waters separating allopatric rearing grounds from SP spawning grounds during most years have been proposed as a migration barrier to adult CV, but not SP splittail, and a mechanism limiting gene transfer between the two populations (Feyrer *et al*., 2015). As the physiological responses of adult splittail to ecologically relevant salinities have not yet been compared between the two populations, here we investigate interpopulation variation in adult physiological responses to salinity exposure. Evidence of local adaptation to elevated salinities throughout the lifecycle of SP splittail may provide opportunities for managing this once federally listed species (United States Fish and Wildlife Service, 1999) under the threat of salinity intrusion to upstream freshwater habitats of the SFE (Knowles and Cayan, 2002; Cayan *et al*., 2008; Cloern and Jassby, 2012), like the splittail sympatric rearing grounds and CV spawning grounds.

**Figure 1:**
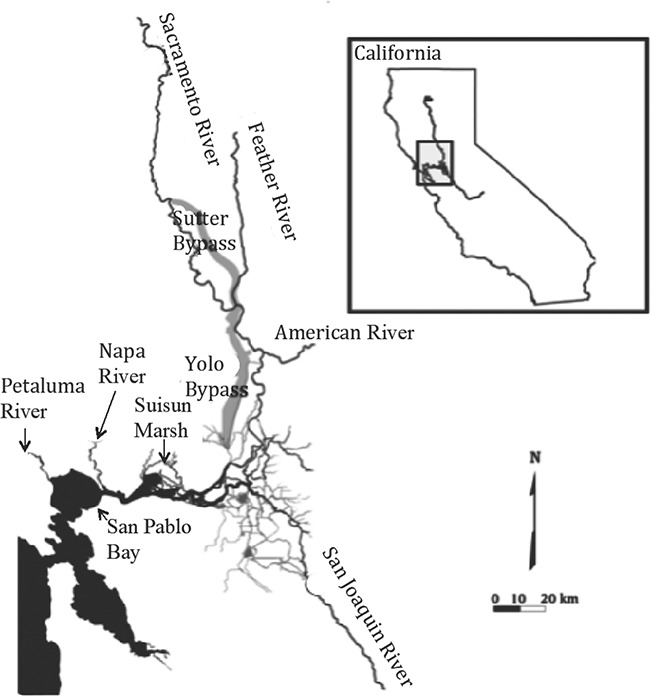
The San Francisco Estuary (in California) and its associated rivers and estuaries with sampling locations and putative spawning locations for the wild San Pablo and Central Valley populations of Sacramento Splittail (*Pogonichthys macrolepidotus*). The putative spawning locations for the San Pablo splittail population are the Napa and Petaluma Rivers. The putative spawning locations for the Central Valley splittail population are Suisun Marsh, the Sacramento and American Rivers and tributaries of the San Joaquin River. Carquinez Strait (not labelled) separates Suisun Marsh and San Pablo Bay. San Pablo splittail collections occurred in Napa and Petaluma Rivers. Central Valley splittail were collected at sites between the confluence of the American and Sacramento Rivers and Suisun Marsh.

**Figure 2:**
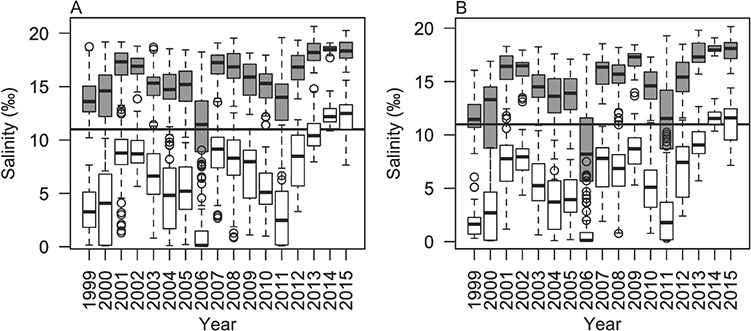
Yearly box plots of daily maximum (grey) and minimum (white) water salinities measured at the upstream entrance to San Pablo Bay (USGS Water Quality Station 11455820 at Carquinez Strait; USGS 2019) during expected adult Sacramento splittail (*Pogonichthys macrolepidotus*) spawning migration (February to April). Salinity monitoring is reported for 14.6 m (**A**) and 1.5 m (**B**) above the bed of Carquinez Strait. Horizontal black lines indicate 11‰ water salinity, the experimental salinity exposure for this study. Upper, middle and lower bands represent the 75^th^, 50^th^ and 25^th^ percentiles, respectively. Whiskers illustrate the range of non-outlier points; more specifically, the most extreme data points that fall no farther than ‘the range times the interquartile range’ from the upper or lower bands of the box. The yearly interquartile ranges of daily maximum water salinity exceed 11‰ for 16 out of 17 years when measured at 14.6 m above the bed and 13 out of 17 years when measured at 1.5 m above the bed.

Highly fecund adult splittail migrate annually between the freshwater to slightly brackish, food-rich rearing grounds of the Upper SFE and Delta to the downstream spawning grounds (Feyrer and Baxter, 1998; Moyle, 2002). The SP and CV populations appear to overlap throughout much of the rearing grounds, while utilizing primarily allopatric spawning habitats (Baerwald *et al*., 2008; Feyrer *et al*., 2015). Age-0 SP splittail are captured exclusively in the relatively brackish Napa and Petaluma Rivers, suggesting that spawning adults must pass from the rearing grounds through the much more saline Carquinez Strait (between Suisun Marsh and San Pablo Bay) to reach the SP splittail spawning habitat (Fig. 1). However, conditions in SP spawning grounds and through Carquinez Strait can vary greatly among seasons and years depending on drought and management of freshwater out from the Sacramento and San Juaquin Rivers. According to USGS water quality monitoring, during the adult splittail spawning migration in winter to early spring (Moyle *et al*. 2004), the interquartile range of maximum daily salinity of water passing downstream through Carquinez Strait and into San Pablo Bay fully exceeded 11 ppt for 13 of the 17 years from 1999 to 2015 (U. S. Geological Survey 2019; Fig. 2). During most years, age-0 CV splittail are captured exclusively in the freshwater floodplains and tributaries of the Central Valley (Daniels and Moyle, 1983; Sommer *et al*., 1997; Moyle *et al*., 2004; Baerwald *et al*., 2007, 2008) located upstream of Carquinez Strait. However, during years with high freshwater outputs from the Sacramento and San Joaquin Rivers that result in low water salinity within the estuary, age-0 CV splittail appear in the Napa and Petaluma Rivers (Feyrer *et al*., 2015; Mahardja *et al*., 2015). Although it is unknown whether SP or CV spawning grounds are optimal for either population, CV splittail appear to spawn downstream of Carquinez Strait only during high water years when salinities experienced during this spawning migration route are low. These trends in age-0 CV splittail presence suggest that the genetic distinction between these two populations may reflect differential salinity tolerance of adults during the spawning migration. Based on migration route salinities and trends in spawning locations, we hypothesized that adult CV splittail require lower estuary salinity levels than adult SP splittail do for successful passage to the Napa and Petaluma River spawning grounds. Therefore, this study tests for inter-population differences in salinity tolerance and metabolic capacity that may explain the absence of CV splittail passage through a relatively high-salinity estuary to access SP spawning habitat.

Fish moving from low- to high-salinity waters, such as adult splittail moving from upstream rearing habitats through the estuary to the SP population spawning grounds, are faced with an osmoregulatory challenge. As environmental waters become increasingly hyperosmotic relative to a fish’s blood, water is lost across the gills, resulting in increased plasma osmolality and reduced muscle moisture (Evans, 2008). Fish can compensate for water loss through drinking saltwater, but the gained ions must be excreted from the body by the gills and kidneys. Ionocytes are the main sites of sodium and chloride excretion in fish gills (Foskett and Scheffey, 1982; Perry, 1997; Evans *et al*., 2005). Within the ionocytes (i.e. chloride cells or mitochondrial-rich cells) and kidneys of teleost fishes, Na^+^-K^+^-ATPase (NKA) establishes the electrochemical gradients driving Na^+^ and Cl^−^ excretion to the environment (Karnaky *et al*., 1976; Perry, 1997; Marshall, 2002). The cellular work required to move excess Na^+^ and Cl^−^ ions against electrochemical gradients inevitably adds to the basic metabolic demands in fish (Smith 1930; Ern *et al*., 2014) and previous studies have interpreted fluctuations in metabolic rate with water salinity changes to reflect fluctuations in osmoregulatory costs (e.g. Rao 1968; Christensen *et al*., 2017).

Basic metabolic demands, which are quantified by measuring standard metabolic rate of quiescent post-absorptive fish (Chabot *et al*., 2016), are combined with maximum metabolic rate to define aerobic capacity. Aerobic capacity is measured as aerobic scope (AS; calculated as standard metabolic rate subtracted from maximum metabolic rate) and represents the metabolic power available to support activity, such as migration to spawning grounds, beyond basic maintenance functions (Fry, 1971). These metabolic processes require mobilization of energy stores, such as glucose. Although elevated standard metabolic rate corresponding with hypo- and hyperosmotic environments has been demonstrated in fish (reviewed in Ern *et al*., 2014), previous studies have failed to identify a consistent relationship between the metabolic costs of osmoregulation and the osmotic gradient between the blood and environmental water (for example: Rao, 1968; Sardella and Brauner, 2008; Ern *et al*., 2014; Christensen *et al*., 2017). These inconsistent findings may in part be due to the capacity for fish to sacrifice water and ion regulation in favour of minimizing standard metabolic rate, as seen in rainbow trout (Rao, 1968), and preserving aerobic capacity. Standard and maximum metabolic rates can also be impacted by indirect influences of water and ion disturbances. For example, restructuring of gill epithelium, aimed at limiting transepithelial water loss during acclimation to hyperosmotic water, can impair maximum metabolic rate by slowing oxygen flux from the environment into the blood (Evans *et al*., 2005). Impaired blood oxygen loading at the gills can be exacerbated by impacts of ionic imbalances on cardiac muscle contraction mechanisms that reduce the capacity to circulate blood (e.g. Neilsen and Gesser, 2001). Furthermore, haemoconcentration caused by water loss can increase the metabolic cost of circulating blood and elevate standard metabolic rate. The literature on fishes is rich with measures of osmoregulatory responses to water salinity (for example: Byrne *et al*., 1972; Beamish *et al*., 1978; Young and Cech, 1996; Scott *et al*., 2007; Haller *et al*., 2014) and measures of metabolic responses to a wide range of environmental variables (for example: Byrne *et al*., 1972; Fry 1971), including salinity (Sardella and Brauner 2008; Christensen *et al*., 2017; Christensen *et al*. 2018). However, osmoregulatory and metabolic responses to water salinities have rarely been assessed concurrently (see Sardella and Brauner, 2008; Christensen *et al*., 2017 for exceptions).

The complex life history and ecosystem interactions of the two splittail populations make conservation and management planning of this species challenging. Evidence of splittail population declines and habitat loss during the late 1900s contributed to a federal listing as threatened under the US Endangered Species Act (U.S. Fish and Wildlife Service, 1999). Ultimately, due to ongoing habitat restoration projects and equivocal evidence of population declines, the ESA listed status of splittail was remanded in 2003; however, splittail retain classification as a species of Special Concern by the California Department of Fish and Wildlife. Despite completed and ongoing habitat restoration projects throughout the SFE, upstream intrusion of salinity from the San Francisco Bay caused by climate change and anthropogenic water diversions (Knowles and Cayan, 2002; Cayan *et al*., 2008; Cloern and Jassby, 2012) continues to pose threats to splittail populations, especially in the form of ecosystem salinity levels exceeding historic norms. Evidence of local adaptation of SP splittail to higher salinity habitats may provide mangers with strategies to conserve this species as saline waters increasingly encroach upon freshwater habitats important to CV splittail.

In this study, based on previously reported interpopulation variation in genetics (Baerwald *et al*., 2007, 2008; Mahardja *et al*., 2015) and spawning habitat (Feyrer *et al*., 2015; Mahardja *et al*., 2015), we hypothesized that, as a result of differing ionic- and osmo-regulatory strategies between adult SP and CV splittail, SFE salinities disproportionately impair CV relative to SP splittail migration through the estuary to spawning grounds within the Napa and Petaluma Rivers. Correspondingly, we predicted that osmotic and ionic disruption would be reduced and more quickly recovered in SP relative to CV splittail experiencing a gradual shift from fresh to saline water. We further predicted that improved osmotic and ionic regulation in SP, relative to CV splittail in saline water, would allow for improved maintenance of cardiorespiratory and musculoskeletal function in SP splittail. Improved maintenance of cardiorespiratory and musculoskeletal function would in turn support the aerobic capacity required for migration, but at the cost of an elevated standard metabolic rate in SP splittail. In order to test these predictions, we quantified (i) salinity tolerance; (ii) disruption of osmoregulatory parameters in response to an acute salinity challenge; and (iii) osmoregulatory disruption and metabolic responses to a combined metabolic and chronic salinity challenge in CV and SP adult splittail. This study was designed to elucidate tradeoffs between osmoregulation and maintenance of aerobic capacity by simultaneously tracking osmoregulatory variables and metabolism during osmotic challenges. Because study subjects of both populations were captured from different natural habitats that vary in biotic and abiotic features, including salinity (Feyrer *et al*., 2015; Mahardja *et al*., 2015), this study does not address the potential that the physiological differences between the populations stem from irreversible early life environmental effects. Despite the potentially confounding early life environmental effects on the observations reported here, insights into the metabolic and osmoregulatory limits of wild-born fish of these two populations that may mechanistically underlie spawning habitat variation will help fisheries managers develop management plans that account for the impacts of an increasingly saline estuary on this species.

## Methods

All splittail measured in these studies were wild-captured at an assumed age of 1 year or more (age 1+) based on size. A total of 117 splittail of average mass 260 (±13) g (mean ± standard error of the mean (±sem)) were caught from Napa and Petaluma Rivers (SP locations) and 114 fish of average mass 510 (±11) g were caught from Central Valley locations (Fig. 2; see Supplementary Section for details). Salinities at capture sites ranged from 0 to 16‰. Though fish size differed between the two populations, the limited availability of these wild fish, in particular members of the smaller SP population, prohibited size matching. However, these size differences are consistent with survey data that previously reported correspondingly smaller fork lengths for wild-captured young-of-the-year SP splittail compared to CV splittail (Baerwald *et al*. 2007), though another study on adults found no population differences in length (Feyrer *et al*., 2015). Although size differences between the two populations may influence physiological and metabolic responses to water salinity exposures, study fish are representative of both populations in the wild at least in some years. After 30 days of captive rearing in freshwater-fed tanks (0.4‰), age 1+ wild splittail were individually marked behind the eye using VI Alpha Tags (Northwest Marine Technology Inc., Shaw Island, WA, USA) and fin clips were collected for genotyping to determine population assignment (see Supplementary Section for details). As some tags were lost during the study, genotyping was repeated on all fish after experimental manipulations to confirm population. Fish were captured from March to June and held in the laboratory for 2 to 4 months before initiating experiments in August.

### Salinity tolerance experiments

#### Range finding tests

Salinity range-finding exposures were performed to assess the highest salinity tolerable by wild age 1+ splittail for a duration of 2 weeks and informed the salinity treatment levels for the *Physiological and Metabolic Response to Salinity Exposure* experiment. Mass of fish used for tests were 408 (±10) g and 247 (±8) g, respectively, for wild-caught CV and SP populations. In a similar range finding test, wild-born juvenile CV and SP splittail tolerated 336 h at 14 and 16 ‰, respectively (Verhille *et al*. 2016). Sacramento splittail of undetermined genotype and 91–121 g mass range tolerated salinities as high as 24‰ for 100 h (Young and Cech, 1996). Based on these reported tolerable salinity ranges for juvenile fish, we performed range finding tests at salinities from 24 to 11‰ (Table 1). Wild-captured fish limited the testable number of salinity increments, and both populations were tested in only the highest tolerable salinity common to the two populations using a resolution of 2‰. Because more SP splittail were available for this testing, tests of higher salinities were performed only on this population.

**Table 1:** Summary of salinities tested for wild adult Sacramento splittail (*Pogonichthys macrolepidotus*) range finding testing.

**Salinity (‰)**	**Population**	**h**
11	CV	>336
11	SP	>336
13	CV	168
16	SP	120
18	CV	96
20	SP	96
24	SP	48

Values under the ‘Hours’ column indicate that the number of hours for which 100% survival, assessed as ability to maintain equilibrium, was maintained among all fish in the tank. Under the population column, SP abbreviates San Pablo population and CV abbreviates Central Valley population

Range-finding tests were performed in 500-L recirculating tanks. Fish were moved from freshwater-supplied (0.4‰) stock tanks into test tanks at least 24 h before testing to allow for adjustment to test tanks. Testing began with a 6-h consistent increase in salinity to the test salinity, while salinity was monitored using a calibrated YSI meter (Model 556MPS, YSI, Yellow Springs, OH, USA). Salinity was manipulated by dissolving aquarium salt (Instant Ocean, Blacksburg, VT, USA) into the tanks. This rate of increase was chosen to reflect the natural tidal cycle experienced in the SFE. Once the recirculation system water reached the test salinity, fish were monitored for loss of equilibrium (LOE) for 336 h or until the first fish within a tank lost equilibrium. Once a fish lost equilibrium, testing for that salinity was terminated, and a new tank of naïve fish was tested at the next lowest salinity (Table 1). Only one tank of fish, regardless of population, was tested per salinity, except at 11‰ (determined as the highest tolerable salinity common to the two populations), where one tank for each population was tested. For example, SP splittail were first tested at 24‰. After the first fish lost equilibrium at 24‰, a new batch of fish was ramped to 20‰ and monitored for LOE and so on until, finally 11‰, was tested. All fish within the 11‰ tank survived 336 h at this salinity without LOE, completing the test. For each salinity and population, 5 to 15 fish were tested. The highest-salinity exposure without LOE within 336 h (11‰) for each population was chosen as the treatment salinity for experiments quantifying effects of a salinity treatment and a metabolic challenge on osmoregulation and metabolism. The fish used for range finding tests were not used for any other experiments.

### Physiological and metabolic responses to salinity exposure

#### Overview

Age 1+ wild-caught splittail of SP and CV populations were used to test the separate and interactive effects of an 11‰ salinity treatment and a metabolic challenge on whole animal-, cellular- and tissue-level ionic and osmotic regulation as well as metabolism and mobilization of metabolic fuels. The 11‰ salinity treatment was chosen as the highest tolerable salinity common to the two populations in the range finding tests and falls within the daily range of salinities occurring in Carquinez Strait during spawning migrations for all years except rare wet years (Fig. 2).

Salinity treatments, lasting for 168 or 336 h, were carried out in six mixed population tanks. Individually identified fish were randomly allocated to tanks 1 week before the initiation of treatments. An additional three control tanks were supplied with freshwater (0.4‰) and stocked with randomly allocated mixed-population fish at the same time. Sampling time points were chosen based on the findings of a similar study of juvenile Sacramento splittail (Verhille *et al*., 2016), suggesting that recovery towards a new homeostasis began between the 168- and 336-h time points, depending on the population and variables used to assess osmotic disturbance. In order to allow sufficient time to perform fish manipulations and measurements at each time point, treatments and sampling were staggered among the tanks, with the entire experiment occurring over a 90-day period. Salinity treatments were planned to provide sample sizes of 14 fish per population per salinity treatment time point. Due to limitations in availability of these wild-captured fish, control sampling of both populations was evenly spread throughout the 90-day study period, with a planned ultimate sample size of 12 control fish per population. Difficulties with sample collection from some fish and equipment failures reduced both control and salinity treatment sample sizes; final sample sizes for all treatment groups are listed in Table S1.

During the 1-week adjustment period between fish allocation to tanks and initiation of salinity exposures, and throughout the entire experiment for fish in control treatment tanks, all tanks within the experimental system were supplied with flow-through 18.5 (±0.5) °C well water. Salinity treatments were initiated by increasing water salinity at a constant rate (1.76‰ hr^−1^) over 6 h and transitioning tank water supply from flow through to recirculation. Daily checks of dissolved oxygen, temperature and nitrogenous waste levels, including ammonia (salicylate test), total nitrite (polyethylene glycol) and total nitrate (polyethylene glycol sulfanilamide) levels (API Fresh and Saltwater Master Test Kits, Mars Inc., McLean, VA, USA) were performed to monitor recirculation system water quality. Feed rations were maintained as in stock tanks, but with a 24-h fast before initiation of sampling and metabolism measurements.

#### Osmotic disturbances

Fish exposed to increasing salinity from 0.4 to 11‰ were sampled before and after a metabolic challenge (described below) at 168- or 336-h salinity exposure and compared with control fish sampled identically throughout the duration of the study. Osmotic and ionic disturbance was assessed through non-terminal blood sampling before and after the metabolic challenge and terminal tissue sampling after the metabolic challenge (Table 2). At each sampling point, fish were netted into a half dose of buffered anaesthetic (0.25 g L^−1^ tricaine methanesulfonate; Argent Inc., Redwood, WA, USA) to achieve light sedation for collection of a small (0.5 mL) blood sample via caudal puncture into a heparinized syringe. Haematocrit, total blood haemoglobin and plasma ion and osmolality measurements were performed on blood samples. When blood volumes were sufficient, plasma glucose and lactate were also measured to quantify effects of salinity treatments on metabolic fuel mobilization. Fish removal from the treatment tank, blood collection and fish placement into the respirometer chamber all occurred within 3 min. In cases where blood was not successfully collected within 2 min after removing the fish from the treatment tank, blood collection was abandoned for that fish, resulting in reduced sample sizes for blood variables associated with RMR measurements.

**Table 2:** Blood and tissue sample collection to track osmotic and metabolic fuel responses to salinity exposure and metabolic challenge for Central Valley and San Pablo populations of the Sacramento splittail (*Pogonichthys macrolepidotus*).

**Variables**	**Pre-metabolic challenge**	**Post-metabolic challenge**
Plasma osmolality	X	X
Plasma ions	X	X
Plasma glucose	X	X
Plasma lactate	X	X
Haematocrit	X	X
Total blood haemoglobin	X	X
Gill NKA		X
Skeletal muscle moisture		X
Ventricular muscle moisture		X

NKA (Na^+^-K^+^-APTase activity). Fish were repeatedly sampled to assess osmotic disturbance, metabolic rate and metabolic fuels at pre- and post-metabolic challenge (four-min swimming/air exposure protocol). All blood sampling was non-terminal, whereas tissue samples were collected from fish euthanized by anaesthetic overdose 1 h after a metabolic challenge. See written methods for more details on the salinity exposure and the metabolic challenge

Total blood haemoglobin was measured for triplicate 10-μL samples of whole blood using a Randox total haemoglobin assay kit (HG980, Randox Laboratories Ltd, Antrim, UK). The whole blood not required for haematocrit or total blood haemoglobin measurements was immediately centrifuged to collect plasma, which was frozen in liquid nitrogen. Plasma glucose and lactate were measured through electrochemical oxidation of hydrogen peroxide in a YSI 2700 biochemistry analyzer (YSI Life Sciences). Plasma chloride was measured in duplicate 10-μL samples using a digital chloridomoter (Labconco, Kansas City, MO, USA), while potassium and sodium measurements were performed, also in duplicate, using 40 and 6 μL of plasma, respectively, in a flame photometer (Model 02655-90, Cole-Palmer Instrument Company, Vernon Hills, IL, USA), which was calibrated as per the manufacturer’s instructions.

Detailed methodologies for measurement of haematocrit and plasma osmolality are provided in Verhille *et al*. (2016). Briefly, haematocrit was measured in duplicate as the percent of packed red blood cell volume in microhaematocrit centrifuge tubes. Plasma osmolality was measured in duplicate using a Vapro™ Vapor Pressure Osmometer (Model 5600) equipped with a mini sample holder to allow for small (2 μL) sample volumes.

For terminal sampling, fish were netted into buffered anaesthetic (0.5 g L^−1^ tricaine methanesulfonate; Argent Inc., Redwood, WA, USA) until becoming unresponsive to a pinch to the caudal peduncle. Fish were humanely euthanized by cervical dislocation then rapidly sampled for mass, fork length, gill, skeletal muscle, ventricular muscle and fin clips. All samples were collected from each individual fish within 10 min after netting into the anaesthetic. Fin clips were collected into vials of 100% ethanol for genotyping to confirm population assignment (see Supplementary Section for genotyping protocols). Detailed methodologies for measurement of muscle moisture and gill NKA activity are provided in Verhille *et al*., (2016). Briefly, gill tissue samples for determination of gill NKA activity were immediately frozen in liquid nitrogen. Gill NKA activity was quantified according to the methods of McCormick (1993) by spectrophotometrically measuring the rate of NADH loss through enzymatically coupling NKA-catalyzed ATP hydrolysis with the oxidation of NADH to NAD catalyzed by lactate dehydrogenase. Skeletal and ventricular muscle moisture were determined as the ratio of wet mass lost with desiccation to total wet mass.

#### Metabolic effects of 168 and 336-h salinity treatments

Metabolic effects of salinity treatments were assessed through measurements of routine metabolic rate (RMR), active metabolic rate (AMR) and plasma glucose and lactate concentrations at rest and immediately after a metabolic challenge. Routine and active metabolic rates were considered estimates of standard and maximum metabolic rate, respectively (Chabot *et al*., 2016). RMR and AMR measurements were performed for freshwater control treatments and at 168- and 336-h salinity treatment time points in conjunction with measurements of osmotic disturbance and metabolic fuel mobilization variables. Aerobic scope (AS) was calculated as the RMR subtracted from AMR.

Intermittent measurements of RMR were performed overnight in static recirculating respirometers. Immediately after sedation and blood sample collection, fish were individually placed into one of four 10-L static recirculating respirometers for overnight automated RMR measurements. Within 1 min after placement into the respirometer, all fish were alert and active in the respirometer, suggesting that the effects of sedation on subsequent RMR measurements were minimized. Oxygen percent saturation of the respirometer water was monitored using one microcathode oxygen electrode per chamber, connected to a 782 Dual-Channel Oxygen Meter (Strathkelvin Instruments, Germany). Oxygen drop (in mg O_2_) was calculated for a minimum 2-min period with the respirometer sealed, during which oxygen levels were never allowed to fall below 80% saturation. To restore oxygen levels in the respirometer, an automated flush pump connected to the external water bath refreshed respirometer water for periods of 5 to 20 min. Water temperature of the external bath was monitored with a YSI dissolved oxygen meter.

Two-point temperature-paired calibrations at 100 and 0% oxygen saturation were performed weekly on the microcathode oxygen electrodes. Background oxygen consumption measurements of all respirometer chambers were performed at the beginning and end of the experiment. No significant oxygen consumption in 11‰ or freshwater was detected for these background biological oxygen demand controls.

Percent saturation was converted to oxygen concentration ([O_2_], mg O_2_ L^−1^) using the following formula:

[O_2_] = % O_2_Sat/100 × α(O_2_) × BP

where %O_2_Sat is the percent oxygen saturation of the water read by the oxygen probes; α (O_2_) is the solubility coefficient of oxygen in water at the water temperature and salinity (mg O_2_ L^−1^ mmHg^−1^); and BP is barometric pressure in mmHg.

Metabolic rate (MR in mg O_2_ kg^−0.95^ min^−1^) was calculated according to the formula:

MR = {[(O_2_(A) − O_2_(B)) × V] × M^−0.95^} × T^−1^_,_

where O_2_(A) is the oxygen concentration in the respirometer at the beginning of the seal (mg O_2_ L^−1^); O_2_(B) is the oxygen concentration in the respirometer at the end of the seal (mg O_2_ L^−1^); V is the volume of the respirometer with the fish volume subtracted from it (L); M is the mass of the fish (kg); and T is the duration of the seal (min). To account for individual variation in body mass, RMRs were allometrically corrected for fish mass using the exponent 0.95. This value is halfway between the life-stage-independent exponent determined for resting (0.97) and active (0.93) zebrafish (Lucas *et al*. 2014); we are aware of no determinations of allometric coefficients for Sacramento splittail.

A metabolic challenge protocol was designed to quantify AMR and osmoregulatory status of fish stimulated to maximum aerobic capacity. The metabolic challenge involved a 3-min chase followed by a 1-min air exposure according to the protocol described by Clark *et al*., (2013). Post-metabolic challenge measurements of AMR were performed in a static respirometer by tracking metabolic rate immediately after the metabolic challenge using intermittent respirometry. The morning after overnight RMR measurements, each fish was removed from the respirometer and placed into a 100-L chase tank filled with treatment water maintained at 15 to 19°C and >80% oxygen saturation. The chase tank was covered with an opaque insulation wrap in order to minimize temperature fluctuations and stress to the fish. After a 30-min adjustment period, the chase tank cover was removed and the fish was chased for 3 min. Chasing involved lightly touching the caudal fin repeatedly to stimulate the fish to swim. During the 3-min chase, all fish exhibited two behavioural phases: vigorous bursts, usually lasting ca. 30 s, followed by constant moderate swimming. The 3-min chase was followed by a 1-min air exposure, during which fish were sufficiently exhausted to allow collection of a 0.5-mL blood sample without anaesthetic.

Although all metabolic rates were allometrically corrected to account for the different sizes of SP and CV fish (exponent 0.95; Lucas *et al*. 2014), the allometric exponent specific to splittail has not been derived and the small sample sizes here precluded its derivation with this study data. In order to avoid misinterpretation of data caused by incorrect allometric adjustments for size, we focused on comparing metabolic responses to salinity exposures within a population rather than direct comparisons of metabolic rate and aerobic scope magnitudes between the two populations.

Measurements of post-metabolic challenge total blood haemoglobin, haematocrit and plasma osmolality and ions (sodium, chloride and potassium) and metabolic fuels were performed on these blood samples (Table 2).

Immediately following the metabolic challenge, fish were placed into a 40-L recovery respirometer, and post-metabolic challenge metabolic rate was tracked using intermittent respirometry for 1 h. The respirometry chamber was partly submerged in a water bath filled with 18.5 (±0.5) °C-regulated water to stabilize respirometer water temperature and equipped with a recirculation pump to maintain water circulation. Water oxygen saturation was monitored using a dipping probe mini oxygen sensor, connected to AutoResp software through a four-channel Witrox oxygen meter (Loligo® Systems, Denmark). Probes were calibrated following the same protocol as with the microcathode oxygen electrodes used for RMR measurements, accounting for salinity differences between treatments. The drop in oxygen saturation was monitored for a 10-min period when the respirometer was sealed without allowing oxygen levels to fall below 80% saturation. To restore respirometer water oxygen saturation to 100%, a valve-controlled tap supplying aerated experimental treatment water to the respirometer was opened, allowing water to overflow out the standpipe for periods of 6 min.

Metabolic rate was calculated and allometrically adjusted as described for RMR measurements. Metabolic rates were calculated for every 2 min interval of O_2_ measurements performed during the one-hour recovery period; 2 min was the minimum interval that allowed for calculation of slope in O_2_ over time with *r*^2^ values ≥0.9 (Hvas and Oppedal, 2019; Zhang *et al*., 2019). Active metabolic rate was determined as the highest value recorded over 2 min during the 1-h recovery period after the metabolic challenge. After a 1-h recovery period from the metabolic challenge, fish were netted out of the respirometer into anaesthetic for terminal sampling to collect tissue samples for measurement of skeletal and ventricular muscle moisture and gill NKA activity (Table 2).

### Statistical analysis

The individual and interactive effects of population, the salinity treatment duration and the metabolic challenge on most osmoregulatory variables were investigated using mixed effects ANOVAs with individual fish as a random effect followed by pairwise comparisons on estimated marginal means determined from the model parameters. As skeletal and ventricular muscle moisture and gill NKA were only sampled once (at terminal sampling) per fish, analyses of these three variables did not include the random effect individual fish. As datasets quantifying effects of 11‰ water treatment duration (0, 168 and 336 h), metabolic challenge (pre-metabolic challenge and post-metabolic challenge), population (SP and CV) and their interactions on dependent variables were unbalanced and non-independently sampled, the lmer function of the lme4 package (Bates *et al*., 2015) in R (R Core Team, 2019) was used. To address the repeated sampling of fish pre- and post-metabolic challenge, ANOVAs included Fish ID as a random effect. In order to perform pairwise comparisons, a reference grid of estimated marginal means, based on the mixed effects ANOVA parameters (Searle *et al*., 1980), was determined using the emmeans function of the emmeans package (Lenth, 2018) in R. Degrees of freedom were estimated using Satterthwaite’s method in the lmerTest package (Kuznetsova *et al*., 2017) in order to estimate *P* values for main effects using Type III sum of squares within the mixed effects ANOVA. Five sets of pairwise comparisons (among salinity treatment duration within metabolic challenge for each population separately, between metabolic challenge within salinity treatment duration for each population separately and between population within salinity treatment duration and metabolic challenge) were then performed for the reference grid of each dependent variable. Pairwise comparisons, which applied *P* values of ≤ 0.05 as the significance cut-off point, employed the Kenward–Roger method for approximation of degrees of freedom and Scheffe’s method for adjustment of *P* values.

Skeletal and ventricular muscle moisture and gill NKA were quantified only one time per fish, so ANOVA testing for treatment effects on these variables did not include the random-effect individual fish. Due to a sample processing error for gill samples collected from fish held at 11‰ for 168 h, gill NKA was only analyzed for samples collected from fish held at 11‰ for 336 h and freshwater controls (ANOVA included only the factors population and salinity with levels: CV and SP and freshwater and 11‰, respectively).

For metabolism measurements and calculated scopes, ANOVAs including the factors population and salinity treatment duration using the lm function of the base R package were used. As metabolic rates and scopes for 168 and 336 h acclimation to 11‰ water were similar (results not shown), the two salinity treatment durations were pooled in the linear model, resulting in a salinity factor (freshwater and 11‰ water) instead of salinity treatment duration. Like for the osmoregulatory variables, pairwise comparisons were performed on the reference grid of estimated marginal means using the Kenward–Roger method for approximation of degrees of freedom and Scheffe’s method for adjustment of *P* values. In order to estimate *P* values based on Type III sum of squares for the dependent variables modelled with the lm function (i.e. skeletal and ventricular muscle moisture, gill NKA and the metabolic rate variables), the contrast coding scheme in R was set to sum contrasts for unordered variables and ANOVAs were performed using the Anova function of the ‘car’ package in R (Fox and Weisberg 2011) with the type parameter set to ‘III’.

## Results

### Genotyping

Of the 117 fish caught in the Napa and Petaluma Rivers, 3% were unidentifiable to population and 8% were assigned to the CV population, with the remaining 89% assigned to the SP population. Of 114 fish caught in Central Valley locations, 4% were unidentifiable to population and only one fish was identified to the SP population, with the remaining 95% assigning to the CV population. Experimental fish were classified to population by genotypes and measurements on all fish that were unidentifiable to population were discarded.

### Salinity tolerance experiments

#### Range finding tests

Though the only salinity test common to CV and SP splittail was 11‰, we did show an overall progression of increasing time to LOE (<48 h to > 336 h, respectively) with decreasing salinity from 24 to 11‰ in splittail (Table 1). 100% of SP and CV splittail endured 20 and 18 ‰, respectively, for 96 h. The highest salinity either population tolerated for a minimum of 336 h was 11‰. As SP splittail were not tested at any salinities between 11 and 16‰, it is not clear where within this range the high tolerance threshold lies for this population. The CV splittail population lost equilibrium by 168 h at 13‰, suggesting that the high tolerance threshold for this population lies between 11 and 13‰. This experiment showed that the maximum salinity tolerable for 336 h was 11‰ for both populations.

### Osmoregulatory responses to the combined salinity and metabolic challenge

ANOVAs showed significant effects of population and salinity on many of the osmoregulatory variables through pre- and post-metabolic challenge sampling. Plasma chloride and sodium and haematocrit and total blood haemoglobin all significantly differed by population (Table 3). Plasma osmolality, chloride, sodium and potassium were significantly influenced by salinity treatments. Only plasma osmolality and chloride were affected by an interaction between population and salinity treatment. Plasma chloride and sodium were significantly affected by an interaction between salinity and the metabolic challenge.

**Table 3:** ANOVA results for analyses of effects of population (Pop), salinity treatment duration (SalHr) and exercise challenge (Swim) on osmoregulatory and metabolic fuel variables in Sacramento splittail (*Pogonichthys macrolepidotus*)

	**df**	***f* value**	***P* value**
**Osmolality (mmol kg** ^**−1**^**)**			
Pop	1	3.6	0.061
SalHr	2	130.6	<0.001
Swim	1	5.6	0.021
Pop *SalHr	2	5.5	0.006
**Chloride (mmol kg** ^**−1**^**)**			
Pop	1	4.5	0.037
SalHr	2	98.5	<0.001
Swim	1	61.2	<0.001
Pop* SalHr	2	5.03	0.009
Swim* SalHr	2	13.9	<0.001
Pop*Swim	1	5.00	0.031
**Sodium (mmol kg** ^**−1**^**)**			
Pop	1	5.9	0.017
SalHr	2	5.4	0.006
Swim	1	11.8	<0.001
Swim* SalHr	2	3.5	0.034
**Potassium (mmol kg** ^**−1**^**)**			
Pop	1	2.8	0.097
SalHr	2	7.5	<0.001
Swim	1	31.4	<0.001
**Haematocrit (%)**			
Pop	1	1.2	0.002
SalHr	2	0.8	0.436
Swim	1	9.1	0.004
**Haemoglobin (g dL** ^**−1**^**)**			
Pop	1	8.2	0.005
SalHr	2	0.3	0.742
Swim	1	0.5	0.476
Swim* SalHr	2	2.5	0.089
**Glucose (g L** ^**−1**^**)**			
Pop	1	1.0	0.319
SalHr	2	22.7	<0.001
Swim	1	41.4	<0.001
Swim* SalHr	2	23.3	<0.001
**Lactate (g L** ^**−1**^**)**			
Pop	1	2.2	0.147
SalHr	2	11.3	<0.001
Swim	1	199.6	<0.001
Swim* SalHr	2	7.7	0.003
**Skeletal Muscle (%)**			
Pop	1	0.1	0.745
SalHr	2	1.5	0.227
**Ventricular Muscle (%)**			
Pop	1	7.1	0.010
SalHr	2	0.6	0.557
**Gill NKA** (**μmol ADP mg protein**^**−1**^ **h**^**-1)**^			
Pop	1	0.9	0.354
SalHr	1	0.2	0.653

NKA (Na^+^-K^+^-APTase activity). All variables were categorical factors, with Pop levels of San Pablo and Central Valley; SalHr levels of freshwater control, 11‰ for 168 h and 11‰ for 336 h; and Swim levels of pre-metabolic challenge and post-metabolic challenge. Although all possible interactions were included in the analyses, only interactions with *P* values<0.1 are shown

ANOVAs investigating cellular and tissue-level (haematocrit, haemoglobin, gill NKA and skeletal and ventricular muscle moisture) responses to salinity treatments did not reflect the salinity-induced physiological disruption seen in plasma osmolality and ions (Table 3).

#### Osmoregulatory responses to salinity pre-metabolic challenge

**Figure 3:**
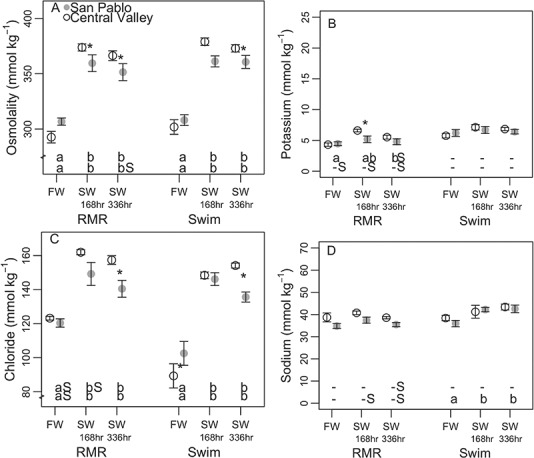
Plasma osmolality (mean ± sem) (**A**), potassium (**B**), chloride (**C**) and sodium (**D**) of adult Central Valley (CV) and San Pablo (SP) population Sacramento splittail (*Pogonichthys macrolepidotus*) sampled after acclimation to 11‰ (SW) water for 168 or 336 h and in 0.4‰ (FW) water. Fish were repeatedly sampled to assess osmotic disturbance pre- (RMR) and post-metabolic challenge (Swim; swimming/air exposure protocol). Letters and asterisks symbolize results from multiple comparisons using significance cut-offs of *P* values ≤0.05. Lowercase letters along the bottom of plots symbolize significant differences between salinity treatments within population and metabolic challenge groupings, with the upper row of letters corresponding to the CV population and the lower row corresponding to the SP population. Dashes signify that multiple comparisons showed no significant differences within that grouping. Uppercase ‘S’s signify significant differences between pre- and post-metabolic challenge values for the same population and salinity treatment. Asterisks next to data points signify significant differences between populations for the same salinity and metabolic challenge treatment

Plasma osmolality, potassium, chloride and sodium all increased in magnitude with increasing salinity in pre-metabolic challenge splittail, but increases were not significant for all variables for SP and CV fish (Fig. 3). Osmolality significantly increased by 168 h in 11‰ water and remained significantly elevated relative to freshwater values until 336 h for both populations (Fig. 3A). Where increases with salinity were significant, the magnitude of increase was always greater for CV than SP fish. At both 168 and 336 h in 11‰ water, CV osmolality was significantly greater than SP osmolality.

Elevated salinity resulted in inter-population variation in ionic disturbance, apparent in plasma potassium and chloride. Both increased in magnitude within 168 h in 11‰ water, and significantly so for all but SP plasma potassium (Fig. 3B and C). The magnitude of increase was greater for CV than SP, resulting in significantly elevated values for CV, relative to SP plasma potassium at 168 h and plasma chloride at 336 h. Like plasma osmolality, plasma chloride remained significantly elevated relative to freshwater values until at least 336 h in 11‰ water for both populations. Plasma potassium, which never significantly changed with salinity for the SP population, dropped to a level that was significantly indistinguishable from the freshwater values by 336 h in 11‰ water for CV fish. Although the trends in plasma sodium response to 11‰ water reflected the trends in plasma osmolality for both populations, this variable never significantly changed between freshwater and 11‰ water values (Fig. 3D).

**Figure 4:**
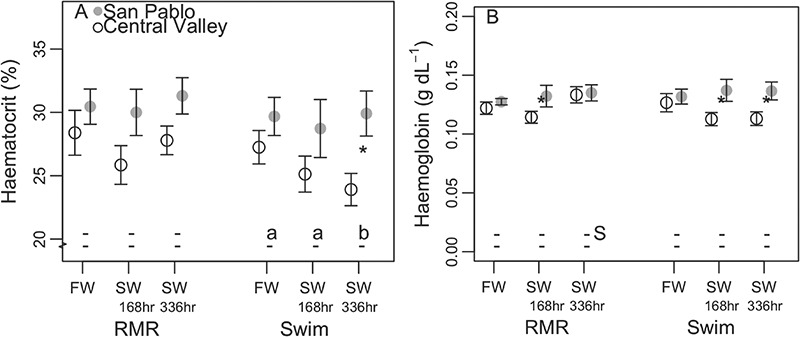
Plasma hematocrit (mean ± sem; A) and haemoglobin (B) of adult Central Valley (CV) and San Pablo (SP) population Sacramento splittail (*Pogonichthys macrolepidotus*) sampled after acclimation to 11‰ (SW) water for 168 or 336 h and in 0.4‰ (FW) water. Fish were repeatedly sampled to assess osmotic disturbance pre- (RMR) and post-metabolic challenge (Swim; swimming/air exposure protocol). Letters and asterisks symbolize results from multiple comparisons using significance cut-offs of *P* values ≤ 0.05. Lowercase letters along the bottom of plots symbolize significant differences between salinity treatments within population and metabolic challenge groupings, with the upper row of letters corresponding to the CV population and the lower row corresponding to the SP population. Dashes signify that pairwise comparisons showed no significant differences within that grouping. Uppercase ‘S’s signify significant differences between pre- and post-metabolic challenge values for the same population and salinity treatment. Asterisks next to data points signify significant differences between populations for the same salinity and metabolic challenge treatment.

The significant disruptions of osmotic and ionic balance, which were often significantly greater for CV than SP splittail, were not reflected in haematocrit, total blood haemoglobin (Fig. 4), skeletal or ventricular muscle moisture or gill NKA activity (Table 3). Although CV splittail haemoglobin values were significantly lower than SP splittail values after 168 h in 11‰ water, values never significantly changed relative to freshwater values for either population (Fig. 4B).

#### Osmoregulatory responses to combined salinity and metabolic challenge

For fish under control conditions (freshwater), the metabolic challenge caused ionic, but not osmotic, disruption. Following the metabolic challenge, plasma potassium increased in magnitude for both populations in freshwater relative to pre-metabolic challenge levels; this change was only significant for SP splittail (Fig. 3B). Plasma chloride decreased in freshwater post-metabolic challenge fish, and this decrease was significant for both populations (Fig. 3C). Neither plasma sodium nor plasma osmolality were influenced by the metabolic challenge for either population in freshwater (Fig. 3D).

The metabolic challenge at 11‰ caused ionic disruption in both populations and some signs of osmotic disruption in SP splittail. Plasma potassium and sodium increased with metabolic challenge in fish of both populations acclimated to 11‰ (Fig. 3B and D), and plasma chloride decreased only for SP splittail (Fig. 3C). As a result, osmolality increased for SP, but not CV splittail with the metabolic challenge and only after 336 h in the 11‰ treatment (Fig. 3A).

Although within population comparisons between pre- and post-metabolic challenge suggest ionic, but not osmotic, disturbance in CV splittail, comparisons of the two populations under the same treatments conditions post-metabolic challenge suggest that CV splittail in fact experience greater osmotic disturbance than SP splittail.

### Metabolic responses to combined salinity and metabolic challenge

ANOVAs showed RMR and AMR to significantly differ between salinity treatments, but not populations (Table 4). On the other hand, AS significantly differed between populations, but not between salinity treatments (Table 4). As size significantly differed between SP and CV splittail, we focused on comparing responses to salinity exposures within a population rather than direct comparisons of metabolism and aerobic scope magnitudes between the two populations.

**Table 4:** ANOVA results for analyses of effects of population (Pop) and salinity treatment duration (SalHr) on metabolic rates and scopes in Sacramento splittail (*Pogonichthys macrolepidotus*)

	**df**	***f* value**	***P* value**
**RMR (mg O**_**2**_ **kg**^**-0.95**^ **min**^**−1**^**)**			
Pop	1	1.7	0.202
SalHr	1	8.5	0.005
**AMR (mg O**_**2**_ **kg**^**-0.95**^ **min**^**−1**^**)**			
Pop	1	0.4	0.509
SalHr	1	8.6	0.005
**AS (mg O**_**2**_ **kg**^**-0.95**^ **min**^**−1**^**)**			
Pop	1	6.1	0.018
SalHr	1	0.6	0.451

All variables were categorical factors, with Pop levels of San Pablo and Central Valley; SalHr levels of freshwater control, 11‰ for 168 and 336 h. Although all possible interactions were included in the analyses, only interactions with *P* values<0.1 are shown. RMR: routine metabolic rate; AMR: active metabolic rate; AS: aerobic scope

**Figure 5:**
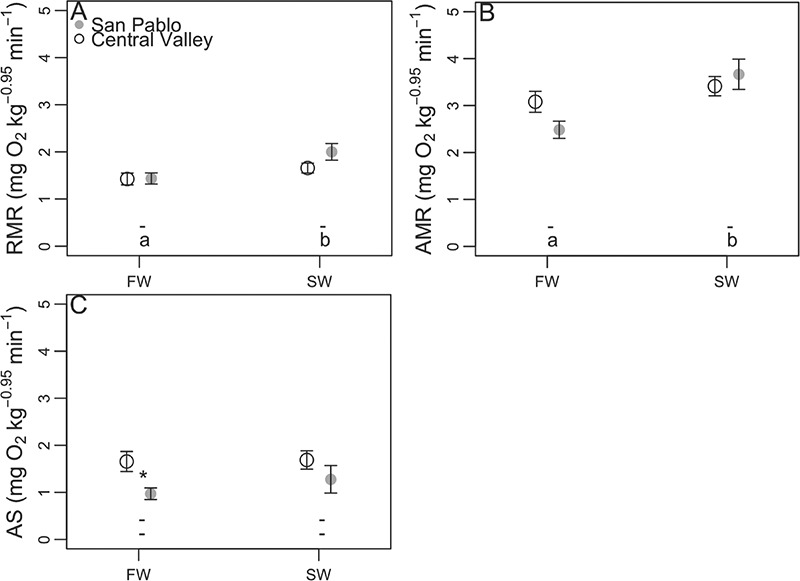
Routine metabolic rate (RMR; mean ± sem; **A**), active metabolic rate (AMR; **B**), and aerobic scope (AS; C) of Central Valley (CV) and San Pablo (SP) populations of Sacramento splittail (*Pogonichthys macrolepidotus*) sampled after acclimation to 11‰ (SW) water or in control 0.4‰ (FW) water. Values for fish acclimated to 11‰ water for 168 or 336 h are pooled with populations maintained separate. Letters and asterisks symbolize results from pairwise comparisons using significance cut-offs of *P* values ≤ 0.05. Lowercase letters along the bottom of plots symbolize significant differences between salinity treatments within the population, with the upper row of letters corresponding to the CV population and the lower row corresponding to the SP population. Dashes signify that multiple comparisons showed no significant differences within that grouping. Asterisks next to data points signify significant differences between populations at the same salinity.

The salinity effect on RMR and AMR was most likely driven by a significant increase in magnitude of both for SP splittail, but not CV splittail. Pairwise comparisons showed RMR and AMR to significantly increase for SP, but not CV fish, in 11‰ relative to in freshwater (Fig. 5A and B).

The population effect on AS was likely driven by significantly greater AS in CV splittail relative to SP splittail in freshwater (Fig. 5C), but may also be an artefact of the different sizes of CV and SP fish. Pairwise comparisons showed that SP splittail maintained a consistent AS as water salinity was increased (Fig. 5C) despite the increase in RMR with increased salinity (Fig. 5A). A significant increase in AMR matching increased RMR with salinity treatment explains the stable AS across salinities for this population.

Plasma glucose and lactate levels, which can be indicative of the fish’s capacity to mobilize energy stores to fuel metabolic processes, were quantified to further describe effects of salinity and metabolic challenge on metabolic processes within SP and CV splittail. ANOVAs showed no significant differences in plasma glucose or lactate between populations but significant effects of salinity duration, metabolic challenge and the interaction between salinity duration and metabolic challenge (Table 3). Notably, for many SP fish pre-metabolic challenge in freshwater, insufficient blood was collected to perform plasma glucose or lactate measurements; therefore, SP splittail glucose and lactate for the freshwater pre-metabolic challenge condition were not included in the statistical analysis.

**Figure 6:**
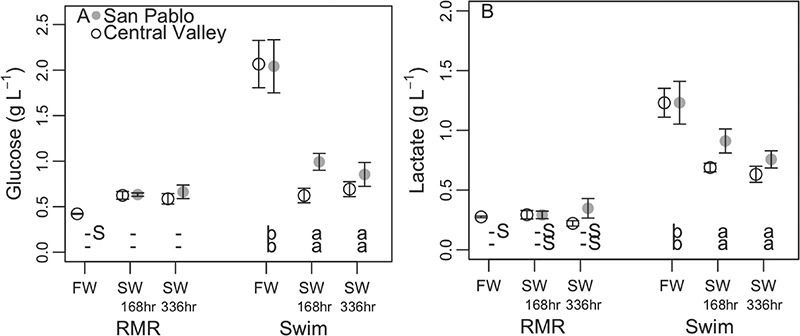
Plasma glucose (mean ± sem; **A**) and lactate (**B**) of adult Central Valley (CV) and San Pablo (SP) population Sacramento splittail (*Pogonichthys macrolepidotus*) sampled after acclimation to 11‰ (SW) water for 168 and 336 h and in 0.4‰ (FW) water. Fish were repeatedly sampled to assess osmotic disturbance pre- (RMR) and post-metabolic challenge (Swim; 4-min swimming/air exposure protocol). Pre-metabolic challenge sampling was non-terminal sampling of lightly sedated fish immediately out of treatment tanks. Post-metabolic challenge blood sampling was of non-anaesthetized fish immediately after a metabolic challenge. Letters and asterisks symbolize results from pairwise comparisons using significance cut-offs of *P* values ≤ 0.05. Lowercase letters along the bottom of plots symbolize significant differences between salinity treatments within population and metabolic challenge groupings, with the upper row of letters corresponding to the CV population and the lower row corresponding to the SP population. Dashes signify that multiple comparisons showed no significant differences within that grouping. Uppercase ‘S’s signify significant differences between pre- and post-metabolic challenge values for the same population and salinity treatment. Asterisks next to data points signify significant differences between populations for the same salinity and metabolic challenge treatment.

Pairwise comparisons showed nearly identical trends in glucose and lactate responses to salinity acclimation, the metabolic challenge and the combined salinity metabolic challenge for SP and CV splittail. However, neither of these two variables were quantified for pre-metabolic challenge SP splittail in freshwater (Fig. 6). Although salinity treatments had no effect on either plasma glucose or lactate of CV splittail pre-metabolic challenge, the metabolic challenge in freshwater resulted in a dramatic and significant increase (4.9-fold for plasma glucose and 4.4-fold for plasma lactate of CV splittail) in both variables. In 11‰ water, the metabolic challenge also resulted in significant increases in plasma lactate, but with an attenuated response compared to in freshwater (from 2.3- to 2.9-fold for CV splittail and from 2.2- to 3.2-fold for SP splittail). There was no significant response of plasma glucose to the metabolic challenge in 11‰ water despite showing similar trends to lactate with salinity and metabolic challenge. As a result of the suppressive effect of salinity on plasma glucose and lactate responses to the metabolic challenge, both variables were significantly higher for CV splittail post-challenge in 11‰ relative to in freshwater.

## Discussion

Two previous companion studies showed improved salinity tolerance in juvenile SP splittail relative to juvenile CV splittail through modestly reduced osmotic disruption to physiological variables (Verhille *et al*., 2016) and increased transcriptome plasticity suggestive of gill remodelling responses to salinity (Jeffries *et al*., 2019) for SP relative to CV splittail. As the temporal and spatial variability in habitat use and energetic demands of spawning adult splittail differ from those of juveniles, here we tested for inter-population variation for adult splittail. This first comparison of salinity effects on wild-born adult CV and SP Sacramento splittail osmoregulatory and metabolic physiology showed evidence of variation in metabolic and osmoregulatory responses between these two populations. It is important to note that study subjects were captured from natural habitats, which are known to vary in biotic and abiotic features, including salinity (Feyrer *et al*., 2015; Mahardja *et al*., 2015), for the two populations. Therefore, further study will be required to elucidate whether inter-population variation in physiology reported here stems from genetic or early life environmental effects. Regardless, this variation was consistent with our prediction of reduced osmotic and ionic disruption in adult SP relative to CV splittail in saline water resulting in maintenance of SP cardiorespiratory and musculoskeletal function to support the high active metabolic rates required for migration through the brackish SFE.

### Salinity tolerance experiments

Salinity range-finding experiments showed that the salinity tolerance of wild adult CV and SP splittail was lower than the tolerances found in a similar study on juvenile splittail. Although the previous study on juvenile splittail involved larger sample sizes, exposures to experimental salinities for 336 h, showed a maximum tolerable salinity of 11‰ for both CV and SP adult splittail (Table 1), whereas juvenile splittail from both populations were able to tolerate 14‰, for over 336 h (Verhille *et al*., 2016). Ontological variation in salinity tolerance (Beamish *et al*., 1978; McCormick 1994; Allen *et al*., 2011; Komoroske *et al*., 2014) and other environmental tolerances (Kelly *et al*., 2014; Komoroske *et al*., 2014) are common in wild fishes with variation generally corresponding to ontological variation in habitat (Varsamos *et al*., 2005; Allen *et al*., 2011; Kelly *et al*., 2014; Komoroske *et al*., 2014). In another previous study (Young and Cech, 1996), slightly smaller (91 to 120 g) wild splittail captured in reputed CV population locations were assessed for time to LOE at salinities ranging from 20 to 28‰. Young and Cech found these splittail to tolerate 20‰ for 72 h, compared to 96 h tolerance of 20‰ for adult SP splittail and 18‰ for adult CV splittail here.

### Osmoregulatory responses to salinity

Osmoregulatory variables (Figs 3 and 4) reflected ranges reported in previous studies, with some discrepancies, likely accounted for through sampling protocols. Plasma osmolality values for freshwater controls of both populations here (range: 280 to 332 mmol kg^−1^; Fig. 3) were similar to mean values reported for juvenile Sacramento splittail (~300 mmol kg^−1^). Additionally, mean values for juveniles at 11‰ (310 to 330 mmol kg^−1^; Verhille *et al*., 2016) fell within the range observed here for wild adults in 11‰ water (314 to 409 mmol kg^−1^). The ranges of haematocrit values observed here for splittail pre-metabolic challenge in freshwater here (27–31%) were lower than values reported for juvenile wild CV and SP splittail (40–45%; Verhille *et al*., 2016) and wild-caught age-0 splittail (37 ± 6.5%; Danley *et al*., 2002). Although the low haematocrit values measured in the adult fish here, relative to in other studies on splittail, could reflect poor health, fish fed normally and no mortalities or other visual signs of poor health were observed in any treatment or control tank over the 90-day experimental period. Mean skeletal muscle moisture values reported for wild juvenile SP and CV splittail (0.72 to 0.76; Verhille *et al*., 2016) fell within the range reported here (0.67 to 0.78) for adult splittail in freshwater. Mean gill NKA values reported for wild juvenile CV and SP splittail (ca. 0.3–0.5 μmol ADP mg protein^−1^ h^−1^; Verhille *et al*., 2016) fell within the rage measured here for adult CV and SP splittail in freshwater (0.23 to 4.84 μmol ADP mg protein^−1^ h^−1^).

The responses of adult Sacramento splittail osmoregulatory variables to an environmental salinity treatment, as seen in pre-metabolic challenge sampling (Figs 3 and 4) showed osmotic and ionic disruptions similar to those of juvenile Sacramento splittail (Verhille *et al*. 2016) and as expected across fish taxa (Byrne *et al*., 1972; Beamish *et al*., 1978; Scott *et al*., 2007; Haller *et al*., 2014). Pairwise comparisons showed that osmolality significantly increased between freshwater controls and 11‰ treatments and remained high after 336 h at 11‰ for fish of both populations. Elevated plasma osmolality with salinity treatment was reflected in all measured plasma ions, but, for plasma sodium, the increase was not significant. However, consistent gill NKA activity across salinity treatments and populations did not correspond with previously reported trends for juveniles Sacramento splittail (Verhille *et al*. 2016; Jeffries *et al*. 2019). Unresponsiveness of haematocrit, total blood haemoglobin and skeletal and ventricular muscle moisture suggest significant cellular or tissue water loss was also not experienced for either population (Sardella and Kültz, 2009).

We predicted improved osmotic and ionic regulation in SP splittail, relative to CV splittail, in saline water. In accordance with this prediction and findings for juvenile Sacrament splittail (Verhille *et al*., 2016), population effects on pre-metabolic challenge plasma osmolality, chloride and potassium are suggestive of a greater osmotic and ionic disturbance in CV relative to SP splittail exposed to salinity treatments. Specifically, plasma osmolality, chloride and potassium disruptions relative to freshwater control values were greater for salinity-treated CV than SP fish with pairwise comparisons showing significant elevations in CV relative to SP fish at many sampling points (Fig. 3).

Although inter-population variation in ionic and osmotic disturbances described here may appear modest, other studies have shown similar osmotic and ionic disruptions and related them to biologically significant changes in whole animal performance. Perch (*Perca fluviatilus*) plasma osmolality increased by only ca. 10 mOsm kg^−1^ with a 10-unit increase in water salinity; this osmoregulatory disruption, which is similar in magnitude as reported in our study, corresponded with a ca. 21% decrease and maximum metabolic rate and a ca. 24% decrease in aerobic scope (Christensen *et al*. 2017). In another study, salt water-acclimated European seabass (*Dicentrarchus labrax*) were able to maintain consistent metabolic rate, cardiovascular function and swimming capacity but could not survive anaesthetization when transferred from salt to freshwater and experiencing a ca. 10 mOsm kg^−1^ plasma osmolality disruption (Chatelier *et al*. 2005). Blood samples collected from radiotelemetry-tagged adult sockeye salmon before river entry during their spawning migration were analyzed to relate blood biochemistry with success at river entry and reaching natal spawning grounds. Fish that successfully entered rivers were significantly distinguished from failures by a mere 5 to 11 mmol kg^−1^ plasma Na content, depending on the stock. Additionally, fish that failed to reach the fish spawning grounds were significantly distinguished from successful fish by only 9 to 22 mOsmol kg^−1^ (Cooke *et al*. 2006).

### Osmoregulatory response to a combined salinity and metabolic challenge

Effects of the metabolic challenge alone, as assessed by comparing pre- and post-metabolic challenge measurements in freshwater, provoked ionic, but not osmotic, disruption in both splittail populations (Figs 3 and 4). The metabolic challenge in freshwater caused significant reductions in plasma chloride and increases in plasma potassium, but no effect to overall plasma osmolality. This response in Sacramento splittail was exaggerated compared to in Atlantic salmon (*Salmo salar*), which experienced little change to plasma potassium, chloride or osmolality after a 2-h swim in freshwater (Byrne *et al*., 1972), although plasma potassium of sockeye salmon swum to failure in freshwater increased (Steinhausen *et al*., 2008).

The lack of haematocrit response to the metabolic challenge in freshwater differed from previously reported increases to haematocrit in response to swimming for the same species. Danley *et al*. (2002) reported haematocrit values of ca. 47.5 ± 50 for Sacramento splittail immediately after fish were swum in a flume. These values compared to values of 25.1 ± 1.4 for post-metabolic challenge CV fish. Therefore, in this study, haematocrit was both consistently lower in magnitude and less responsive to a metabolic challenge in freshwater relative to previous findings. The relatively low haematocrit values reported here for fish post-metabolic challenge may be due to reductions to whole animal blood volumes caused by repeat blood sampling; however, this discrepancy in haematocrit magnitude also existed for pre-metabolic challenge sampling, which was the first sample drawn from these fish.

Effects of salinity on fish osmoregulatory responses to the metabolic challenge were quantified by comparing variables pre- and post-metabolic challenge in 11‰ water and showed subtle variation between the two populations (Figs 3 and 4). The trends in CV splittail osmoregulatory responses to the metabolic challenge in 11‰ water were similar to those observed in freshwater, where ionic balance was disrupted but overall osmotic balance was maintained. For SP splittail metabolically challenged in 11‰ water, overall osmotic disruption occurred with ionic disruption. Combining evidence of reduced ionic and osmotic disruption for SP relative to CV splittail with salinity exposure alone with evidence of osmotic disruption in SP, but not CV splittail, after a combined salinity challenge and metabolic challenge suggests differential metabolic costs of osmoregulation between the two populations. Therefore, metabolic responses to the same osmotic challenge were compared between SP and CV splittail.

### Metabolic response to a combined salinity and metabolic challenge

We are unaware of any previously reported metabolism measurements, nor measurements of salinity effects on metabolism for Sacramento splittail. Reports of metabolic responses to water salinity levels for other fish taxa vary and reflect multiple physiological strategies among species faced with an osmotic pressure gradient across the gills (Rao, 1968; Christensen *et al*., 2017). For example, no water salinity impacts were reported on RMR, maximum metabolic rate or aerobic scope of coho salmon smolts (*Oncorhynchus kisutch*; Fang *et al*., 2019); standard metabolic rate or RMR of estuarine red drum (*Sciaenops ocellatus*); Ern and Esbaugh, 2018); or RMR of juvenile estuarine inaga (*Galaxias maculatus*; Urbina and Glover, 2015). On the other hand, water salinity has been shown to impact standard metabolic rate, AMR and AS of perch (Christensen *et al*., 2017); standard metabolic rate and swimming metabolic rate of tilapia (*Tilapia nilotica*; Farmer and Beamish, 1969); and resting metabolic rate of common carp (*Cyprinium carpio*; Ern *et al*., 2014). Fry (1971) refers to salinity as a masking factor, meaning that fish exposed to salinities outside of the isosmotic range must channel available energy if they are to regulate their internal water and ion homeostasis. In fact, early studies on rainbow trout responses to osmotic challenges suggest that fish face a choice between directing energy towards maintenance of internal water and ion homeostasis and allowing internal osmotic and ionic disruption (Rao, 1968). Correspondingly, we predicted that improved osmotic and ionic regulation in SP relative to CV splittail would come at the cost of an elevated RMR. Although RMR of SP and CV splittail never significantly differed in freshwater, we report a significant but modest increase in RMR for SP, but not CV splittail with increased salinity (Fig. 5). Considered alone, the consistent RMR of CV splittail with salinity increase could reflect superior metabolic efficiency and thus salinity tolerance relative to SP splittail. However, when RMR responses to salinity are considered in combination with increased osmoregulatory disturbance in CV relative to SP splittail (Fig. 3), these trends suggest that SP splittail are more plastic in their capacity to immobilize the metabolically demanding processes necessary to osmoregulate in elevated salinity water. This interpretation is consistent with our previously reported findings of divergent transcriptomic responses indicative of improved plasticity in the SP population (Jeffries *et al*., 2019) and provides some evidence supporting our prediction that improved osmotic and ionic regulation in SP, relative to CV splittail in saline water, would allow for improved maintenance of cardiorespiratory and musculoskeletal function in SP splittail.

As previous researchers have demonstrated metabolic effects of water salinity on fish in the absence of altered metabolic rate (e.g. Urbina and Glover, 2015), we also quantified responses of metabolic fuels to water salinity in Sacramento splittail. Water salinity affected juvenile estuarine inaga fuel usage, assessed via ammonia excretion rates and ratios of oxygen consumption to nitrogen excretion, but this signal did not translate into salinity effects on RMR or energy expenditure (Urbina and Glover, 2015). The metabolic fuels, glucose and lactate, have been assessed for Sacramento splittail in response to a metabolic challenge in freshwater, namely swimming in a flume, in one previous study (Danley *et al*., 2002). Control plasma glucose (0.42 ± 0.01 g l^−1^) and lactate (0.28 ± 0.01 g l^−1^) levels of pre-metabolic challenge CV splittail here (Fig. 6) fell within the range of ca. 0.5 to 1.3 g l^−1^ and ca. 0.10 to 0.25 g l^−1^, respectively reported for wild age 0+ splittail by Danley *et al*. (2002). The metabolic challenge here resulted in elevation of plasma glucose levels to 2.07 ± 0.26 g l^−1^, which is contrary to Danley *et al*.’s reported reduction in glucose values to ca. 0.7 ± 0.1 g l^−1^. On the other hand, the increase in plasma lactate to 1.23 ± 0.12 g l^−1^ in response to the metabolic challenge here did reflect that described in the previous study, but levels still remained lower than lactate levels of ca. 0.45 to 0.70 g l^−1^ described by Danley *et al*. (2002).

Although plasma glucose and lactate magnitudes and responses to salinity, metabolic challenge or their combination never significantly differed between CV and SP populations in 11‰ water, the magnitude of glucose and lactate values was consistently greater for SP than CV splittail (Fig. 6). The non-significant but consistently higher plasma glucose and lactate values for SP relative to CV splittail in 11‰ water suggest that SP splittail were not only better able to mobilize metabolic fuels but also possessed a higher capacity than CV splittail to utilize anaerobic pathways to fuel their response to the metabolic challenge. The slightly, although non-significantly, improved capacity of SP relative CV splittail to mobilize metabolic fuels provides modest evidence that the physiological strategies employed by SP and CV splittail faced with a metabolic challenge in saline water differ.

Our prediction that improved osmotic and ionic regulation in SP relative to CV splittail would come at the cost of an elevated RMR was supported by our results. The slightly more robust homeostatic regulation we observed in SP, relative to CV splittail (Figs 3 and 4), appeared to have been metabolically supported by an increase in RMR which was not observed in CV splittail (Fig. 5), with increased water salinity. Increased SP splittail RMR also corresponded with greater mobilization of metabolic fuels (Fig. 6) and AMR (Fig. 5) achieved with a metabolic challenge in saline, relative to in freshwater.

Despite the significant increase in SP splittail RMR with salinity treatment, the corresponding increase in AMR allowed this population to maintain AS in 11‰ water. The capacity to perform aerobic work is quantified by AS and represents the capacity to carry out tasks necessary for survival and reproduction, such as migration, capturing prey and digesting a meal (Fry, 1971). Therefore, although SP splittail appeared to sacrifice low RMR in order to maintain osmotic homeostasis in salt water, improved osmoregulatory status in SP relative to CV splittail potentially allowed SP splittail to improve AMR and thus maintain AS in salt water.

### Summary

Taken together, these metabolic and osmoregulatory data from adult CV and SP splittail support our hypothesis that migration through the SFE estuary to spawning grounds within the Napa and Petaluma Rivers would be more challenging for adult CV than SP splittail and have meaningful implications. We show here that adult SP splittail ionic and osmotic homeostatic regulatory capacity exceeds that of adult CV splittail in an ecologically relevant water salinity challenge. Adult SP splittail homeostatic regulation is supported by an increase in basic metabolic demands with increased water salinity but also results in greater mobilization of metabolic fuels with a metabolic challenge in saline, relative to freshwater. Notably, further study would be required to elucidate whether these population differences for wild-born fish stem from genetic or early life environmental effects. Nevertheless, these findings are consistent with our predictions that improved osmotic and ionic regulation in SP splittail, relative to CV splittail, in saline water will maintain cardiorespiratory and musculoskeletal function to support the high metabolic capacity required for migration and this maintenance of capacity will occur with the cost of an elevated standard metabolic rate. Maintenance of aerobic capacity, despite increased basic demands of homeostatic regulation when faced with seasonally high environmental water salinities, likely equips SP splittail to carry out essential tasks to fitness, such as migration and prey capture. These trends in interpopulation variation support our hypothesis that during most years, SFE salinities present adult CV splittail with a greater challenge than experienced by adult SP splittail when migrating through the estuary to spawning grounds within the Napa and Petaluma Rivers.

Divergent responses between adult splittail populations exposed to salinity manipulations add support to previously reported evidence of local adaptation based on population genetics (Baerwald *et al*. 2008; Mahardja *et al*., 2015) and divergent physiological and transcriptomic responses of juvenile splittail populations to salinity manipulations (Verhille *et al*., 2016; Jeffries *et al*., 2019; Mundy *et al*., 2020). Our studies on juvenile Sacramento splittail showed greater physiological disruption (Verhille *et al*., 2016) and reduced transcriptome plasticity (Jeffries *et al*., 2019; Mundy *et al*., 2020) in CV compared to SP splittail in response to experimental elevation of water salinity. Combined with similar divergent physiological responses reported here for adult splittail, this series of studies suggest that SP splittail are more salinity tolerant relative to CV splittail, throughout their entire life history.

These findings have potentially important management implications. Although Sacrament splittail had briefly been federally listed (United States Fish and Wildlife Service, 1999), current protection status of this sole remaining member of the Pogonichthys genus is limited to only a Species of Special Concern by the California Department of Fish and Wildlife. With salinity intrusion increasingly threatening the upstream historically freshwater habitats (Cloern and Jassby 2012) that CV splittail depend upon, the SP population may become an important reservoir for this species. These adult splittail findings provide further support of the recommendation of population-specific management offered by Verhille *et al*., (2016) based on juvenile splittail findings.

## Supplementary Material

AdultSplitMS_SUPPrev1_SUBMIT_coaa098
