# Supplementary material for: Inter-population differences in salinity tolerance of adult wild Sacramento splittail: osmoregulatory and metabolic responses to salinity
Source: Conserv Physiol. 2020 Dec 10;8(1):coaa098. doi: 10.1093/conphys/coaa098 (PMC7733400; doi:10.1093/conphys/coaa098)
Supplement: AdultSplitMS_SUPPrev1_SUBMIT_coaa098 [file adultsplitms_supprev1_submit_coaa098.docx]

**SUPPLEMENTARY SECTION**

Table S1. Sample sizes for all experimental treatment groups assessing physiological and metabolic responses to salinity exposure

| **Variables** | **Population** | **Pre-metabolic Challenge Sample Size** | | | **Post-metabolic Challenge Sample Size** | | |
| --- | --- | --- | --- | --- | --- | --- | --- |
|  |  | Control | 168 hr | 336 hr | Control | 168 hr | 336 hr |
| Plasma Osmolality | CV | 4 | 8 | 9 | 6 | 11 | 11 |
|  | SP | 9 | 8 | 12 | 15 | 11 | 12 |
| Plasma Cl | CV | 4 | 7 | 7 | 6 | 11 | 11 |
|  | SP | 6 | 4 | 10 | 9 | 11 | 11 |
| Plasma Na | CV | 4 | 9 | 11 | 6 | 11 | 10 |
|  | SP | 7 | 8 | 13 | 10 | 10 | 9 |
| Plasma K | CV | 4 | 9 | 11 | 7 | 11 | 10 |
|  | SP | 7 | 8 | 13 | 9 | 9 | 10 |
| Plasma Glucose | CV | 5 | 8 | 3 | 5 | 8 | 8 |
|  | SP | - | 4 | 5 | 6 | 5 | 9 |
| Plasma Lactate | CV | 5 | 8 | 3 | 7 | 8 | 8 |
|  | SP | - | 4 | 5 | 6 | 6 | 9 |
| Hb | CV | 7 | 13 | 13 | 8 | 11 | 11 |
|  | SP | 11 | 10 | 13 | 10 | 11 | 11 |
| Hct | CV | 7 | 10 | 13 | 8 | 8 | 11 |
|  | SP | 12 | 11 | 13 | 11 | 11 | 11 |
| Gill NKA | CV | - | - | - | 3 | 4 | |
|  | SP | - | - | - | 5 | 4 | |
| Skeletal Muscle Moisture | CV | - | - | - | 10 | 12 | 13 |
|  | SP | - | - | - | 10 | 14 | 13 |
| Cardiac Muscle Moisture | CV | - | - | - | 10 | 10 | 12 |
|  | SP | - | - | - | 10 | 11 | 11 |

SP (San Pablo Sacramento splittail population); CV (Central Valley Sacramento splittail population); Cl (chloride); Na (sodium); K (potassium); Hb (total blood haemoglobin); Hct (haematocrit); NKA (Na^+^-K^+^-APTase activity).

## Wild Fish Capture:

Wild-caught splittail (one year or more in age, age 1+) were gill netted and/or beach seined from the Napa and Petaluma Rivers, Suisun Marsh and the Sacramento-San Joaquin River Delta (Figure 1). Gill nets (2 cm^2^ mesh size) were set for 2 hours, and continuously monitored in order to ensure fish were removed within 2 minutes of entanglement. Beach seine nets were 10-m beach seines with a stretch mesh size of 6 mm.

At the capture site, fish were placed in an aerated cooler filled with capture site water, and then transported to the University of California, Davis (UCD) in a 1000 l aluminum transport tank equipped with an oxygen supply. Transport tank water was maintained below 22 ᵒC and above 80 % oxygen saturation during the trip to UCD.

At UCD, fish were directly transferred into one of two 300 l tanks (i.e. one for each population), supplied with aerated, flow-through, 18.5 ᵒC, degassed well water and held under natural photoperiod lighting. Fish were immediately offered a daily ration of 1.5 to 2.0% body weight of a 1 mm Rangen Salmon feed (Rangen Inc. Snake River Valley, ID, USA). For the first two weeks in captivity, a prophylactic regime of daily 1 ‰ salinity and oxytetracycline (as additive to feed) treatments were administered to prevent disease outbreaks. For the prophylactic salinity treatment, Instant Ocean (Instant Ocean, Blacksburg, VA, USA) was dissolved into the tank water to raise the salinity to 1 ‰, while freshwater flow into the tank was maintained to allow a gradual dilution of salt to fresh water. Fish were held in captivity for a minimum of 30 days before use in any experimental manipulation.

### **Genotyping**

All fish were genotyped to confirm population assignments (i.e., Central Valley or San Pablo population). DNA was extracted from caudal fin tissue using the QIAGEN DNEasy 96 kit (QIAGEN Inc.). A total of eighteen microsatellite markers were amplified for the subsequent population assignment analysis: CypG3, CypG4, CypG23, CypG25, CypG35, CypG39, CypG40, CypG43, CypG45, CypG48, CypG52, CypG53, Pmac1, Pmac4, Pmac19, Pmac24, Pmac25, Pmac35 (Baerwald and May 2004; Mahardja et al. 2012). PCR and allele scoring procedures followed Mahardja et al. (2015). We used STRUCTURE 2.3.3 (Pritchard et al. 2000) to genetically assign individuals to their putative population. Age-0 splittail described in Baerwald et al. (2007) and Mahardja et al. (2015) served as references for the two populations. STRUCTURE analysis was performed for ten iterations at K = 2, with all individuals and references included no prior location information, 500,000 burn-in period, and 1,000,000 Markov chain Monte Carlo repetitions under the assumption of admixture and correlated allele frequencies. Replicate runs were averaged in CLUMPP 1.1.2 (Jakobsson and Rosenberg 2007) with the FullSearch algorithm. Based on the efficiency and accuracy scores found in Vӓhӓ and Primmer (2006), we selected average q-value of 0.8 as the threshold for distinguishing between purebred individuals and potential hybrids/unassigned.

## Supplementary Material References

Baerwald, M. R., and B. May. 2004. Characterization of microsatellite loci for five members of the minnow family Cyprinidae found in the Sacramento–San Joaquin Delta and its tributaries. Molecular Ecology Notes 4:385–390

Baerwald, M. R., Bien, V., Feyrer, F., & May, B. (2007). Genetic analysis reveals two distinct Sacramento splittail (*Pogonichthys macrolepidotus*) populations. *Conservation Genetics*, *8*(1), 159–167. doi:10.1007/s10592-006-9157-2

﻿Jakobsson M., & Rosenberg N.A. (2007). CLUMPP: a cluster matching and permutation program for dealing with label switching and multimodality in analysis of population structure. *Bioinformatics* 23:1801–1806. doi:10.1093/bioinformatics/btm233

Mahardja, B., May, B., & Baerwald, M. R. (2012). Characterization of 36 additional microsatellite loci in splittail (*Pogonichthys macrolepidotus*) and cross-amplification in five other native California cyprinid species. *Conservation Genetics Resources*, *4*, 917–921.

Mahardja, B., May, B., Feyrer, F., & Baerwald, M. R. (2015). Interannual variation in connectivity and comparison of effective population size between two splittail (*Pogonichthys macrolepidotus*) populations in the San Francisco Estuary. *Conservation Genetics*. *16, 385-398*.

﻿Pritchard J.K., Stephens M., & Donnelly P. (2000). Inference of population structure using multilocus genotype data. *Genetics.* 155:945–959

﻿Vähä, J.-P. & Primmer C.R. (2006). Efficiency of model-based Bayesian methods for detecting hybrid individuals under different hybridization scenarios and with different numbers of loci. *Mol Ecol.* 15:63–72. doi:10.1111/j.1365-294X.2005.02773.x
